# Supplementary material for: Geographically dispersed zoonotic tuberculosis in pre-contact South American human populations
Source: Nat Commun. 2022 Mar 7;13:1195. doi: 10.1038/s41467-022-28562-8 (PMC8901693; doi:10.1038/s41467-022-28562-8)
Supplement: Supplementary file 1 — Supplementary Information [file 41467_2022_28562_MOESM1_ESM.pdf]

SUPPLEMENTARY INFORMATION

Contents:

Supplementary Note 1: Provenance and Archaeological Context
Supplementary Figures 1-6
Supplementary References

Supplementary Note 1

Provenance and Archaeological Context

The four samples that yielded data for the reconstruction of three MTBC genomes represent individual 82 buried at the site of Moquegua, M6-Estuquiña located in the junction of the middle and upper Osmore valleys in Peru, and two individuals buried at separate sites in the modern city of Bogotá in Colombia, named Las Delicias (individual 281) and Candelaria La Nueva (individual 386).

Moquegua, M6-Estuquiña, **Peru**
The site of Moquegua, M6-Estuquiña is situated at the juncture of the middle and upper Osmore valleys, ca. 1500 m above sea level [1-3] and is separated by a mountain barrier from the lower valley. M6-Estuquiña is located approximately 65 km inland (by air) from the coast. This site was occupied during the Late Intermediate Period (LIP) (1250-1470 C.E.)[4] by the Altiplano people whose subsistence practices were mainly agrarian [1,2,5,6]. M6-Estuquiña is the type site of the Estuquiña culture, which is distinct from any other inhabiting the Osmore valley [2,4].

Excavations at Moquegua, M6-Estuquiña were carried out in 1985. They were led by the general project director Donald S. Rice (Southern Illinois University) and Jane E. Buikstra (Arizona State University; ASU) who directed the mortuary excavations.

A total of 245 tombs were excavated at Moquegua, M6-Estuquiña, 185 of which had already been opened and disturbed prior to excavation, likely by looters [6,7]. Individual 82 was excavated from Tomb 629, which had previously been disturbed and its burial context left exposed to the surrounding environment. This exposure could account for the high amount of mycobacterial background from soil dwelling mycobacteria present in our sample library from this individual.

Jane E. Buikstra, Sloan Williams and Niki R. Clark carried out mortuary excavations and osteological analyses. Analyses revealed skeletal lesions consistent with prolonged tuberculosis (TB) infection to be prevalent at Moquegua, M6-Estuquiña. Of the investigated individuals 37 had skeletal TB lesions, constituting a minimum of 8.9% of the total population. Amongst adult individuals for which sex could be estimated, 9.8% of females and 19.2% of males displayed skeletal TB lesions affecting vertebrae and/or ribs [7]. This suggests that men

may have been at higher risk of contracting TB, or that they were more likely to sustain a prolonged infection allowing skeletal lesions to form.

Excavations of the residential areas, carried out by Donald S. Rice and Geoffrey W. Conrad revealed that faunal remains were included among the grave goods at the site. Skeletal remains from guinea pigs and llama feet were common finds, as well as the remains of fish, mollusks and birds [2,6,8]. Specifically, the remains of jack mackerel (*Trachurus murphyi*) from the coast have been found in the Moquegua Valley [9]. No pinniped remains were reported.

The advantageous and strategic location of Estuquiña provided access to easily irrigable land, as well as to the corridors coming down from the upper valleys and up from the coast [2]. These corridors were likely used by the llama caravans trading goods between the upper valleys and the coast, an occurrence recorded in historical documents from the 16th century [2,10]. The expansion of the Estuquiña site, which had become a large fortified village by the end of the LIP, may have been the result of its advantageous location[2].

Colombian sites

The archaeological sites of Las Delicias and Candelaria La Nueva are named after the *barrios* (neighborhoods) of Bogotá, Colombia's capital city, in which they are located. Bogotá is situated on the 'Sabana de Bogotá', a plateau in the Eastern Cordillera in the Andes, approximately 2,640 m above sea level and more than 600 km inland from the Pacific coast and 1000 km from the Caribbean coast.

The sites of Las Delicias and Candelaria La Nueva, which are separated by only 3.3 km, were excavated as part of archaeological rescue efforts carried out by researchers at the *Instituto Colombiano de Antropología e Historia* (ICANH). Both sites are located in the territory that belonged to the Muisca confederation, a highly organized coalition of tribes that occupied the area between 950-1550 C.E. until European colonization changed the societal structure [11,12]. The tribes of the Muisca confederation are known to have relied on long-distance trade networks in order to trade salt, emeralds, cotton and clothing for crops cultivated in warmer and lower elevated terrain, such as cocoa, even reaching coastal regions [12-14]. The subsistence practices of the Muisca were primarily agricultural, with high production of corn and beans, supplemented with hunting and freshwater animals caught in lakes and rivers [12,14].

Based on human osteological surveys conducted by Felipe Cárdenas-Arroyo (ICANH), Jane E. Buikstra (ASU), Judith Arnett (University of the Andes) and Laura Paloma Leguizamón (ICANH), only the individuals included in this study displayed skeletal lesions consistent with prolonged TB infection at Candelaria La Nueva (individual 386) and Las Delicias (individuals 281, 387). However, another six individuals from other Muisca territory sites included in this study displayed skeletal lesions characteristic of TB infection – indicating that it was not uncommon in the Muisca confederation, although it has not been recorded at such high prevalence rates as those recorded for Moquegua, M6-Estuquiña. Samples from these six individuals were screened using qPCR and gene-capture methods, but did not yield positive results for MTBC DNA preservation (Supplementary Data 1).

Las Delicias. Braida E. Enciso Ramos BA, led the rescue excavations at Las Delicias. The archaeological site was located on an alluvial terrace of the Tunjuelito River. Five strata were found; one of them (depth: 30 centimeters) contained evidence of human occupation (pers. comm. Laura Paloma Leguizamón). This evidence consisted of tombs, remains of posts from dwellings, evidence of storage in the form of pits, musical instruments crafted from animal bone, charcoal, seeds, pottery, lithics, and animal and human bone remains [15,16]. Archaeological evidence points to this site having been used for domestic, agricultural, and funerary activities. The skeletal remains of at least 18 human individuals were recovered from various tombs at the site [17], along with the remains of deer, birds, fish (likely freshwater) and guinea pigs [16,18]. Paleodemographic analysis revealed that 66.6% of individuals were infants (0 to 4 years) and that adults are highly underrepresented compared to the neighboring site of Candelaria La Nueva where the demography of the cemetery is more equally distributed in terms of age and sex [16].

Radiocarbon dates from the site have been generated from two different types of material. Two dating efforts were commissioned by ICANH from charcoal material. One charcoal sample was associated with a dwelling feature from a domestic context yielding a date of 770 ± 70 C.E. (1180 ± 70 B.P.; Beta-39874). The second charcoal sample was from a pit associated with a grave and yielded a date of 940 ± 70 C.E. (1010 ± 70 B.P.; Beta-39873). Another date (this study) was generated from a sample from a rib from individual 281 (LD-90-1X-011), which yielded a calibrated date of 1265-1380 C.E. (Supplementary Data 11). Based on these radiocarbon dates Las Delicias seems to have been occupied during both the early (950-1250 C.E.) and late (1250- 1550 C.E.) Muisca period [19].

Candelaria La Nueva. Arturo Cifuentes and Leonardo Moreno led the rescue excavations at Candelaria La Nueva, located on a colluvial terrace near the Tunjuelito River [11]. Four strata were identified, one of them (depth: 30-40 centimeters) revealed evidence of human occupation. This evidence consisted of tombs, traces of posts from dwellings, storage traces and pits, charcoal, seeds, pottery, lithics, and animal and human bone remains (pers. comm., 2018 Laura Paloma Leguizamón). Archaeological evidence shows that this site was used for both domestic and funerary activities. 48 disturbed or destroyed human graves were identified at the site, and the skeletal remains of 37 individuals were collected [17]. Paleodemographic analyses revealed that the maximum age for the Candelaria La Nueva population was 45 years, with a high mortality rate among females aged 20-30 years [20]. The faunal remains recovered from the site belong to deer, armadillo, guinea pig, mollusks, and an unidentified feline species [18].

Radiocarbon dating has been carried out for three individuals excavated from Candelaria La Nueva. Two dates were published by Therrien and Enciso [21], using samples from two separate individuals, yielding the following dates: 1250 ± 110 C.E. (700 ± 110 BP; GX-18839-G) and 1175 ± 110 C.E. (775 ±110 BP; GX-18840-G). Radiocarbon dating for individual 386 (87-X-005) (this study) yielded a calibrated date of 1450-1640 C.E. (Supplementary Data 11). These dates indicate that the dated individuals lived during the Muisca period (950-1550 C.E.) [19] and,

potentially in the case of individual 386, overlapped with the arrival of Spanish colonizers in 1536 C.E. [12].

A previous ancient DNA study of samples from 14 of the individuals excavated from Candelaria La Nueva sought to determine the mitochondrial haplogroup of the individuals via Restriction Fragment Length Polymorphism (RFLP) analysis [22]. All ancient individuals were found to carry haplogroup A, a common haplogroup in the Americas, including individual 386 (87-X-005) from whom an *M. pinnipedii* genome was reconstructed in this study.

Supplementary Figures

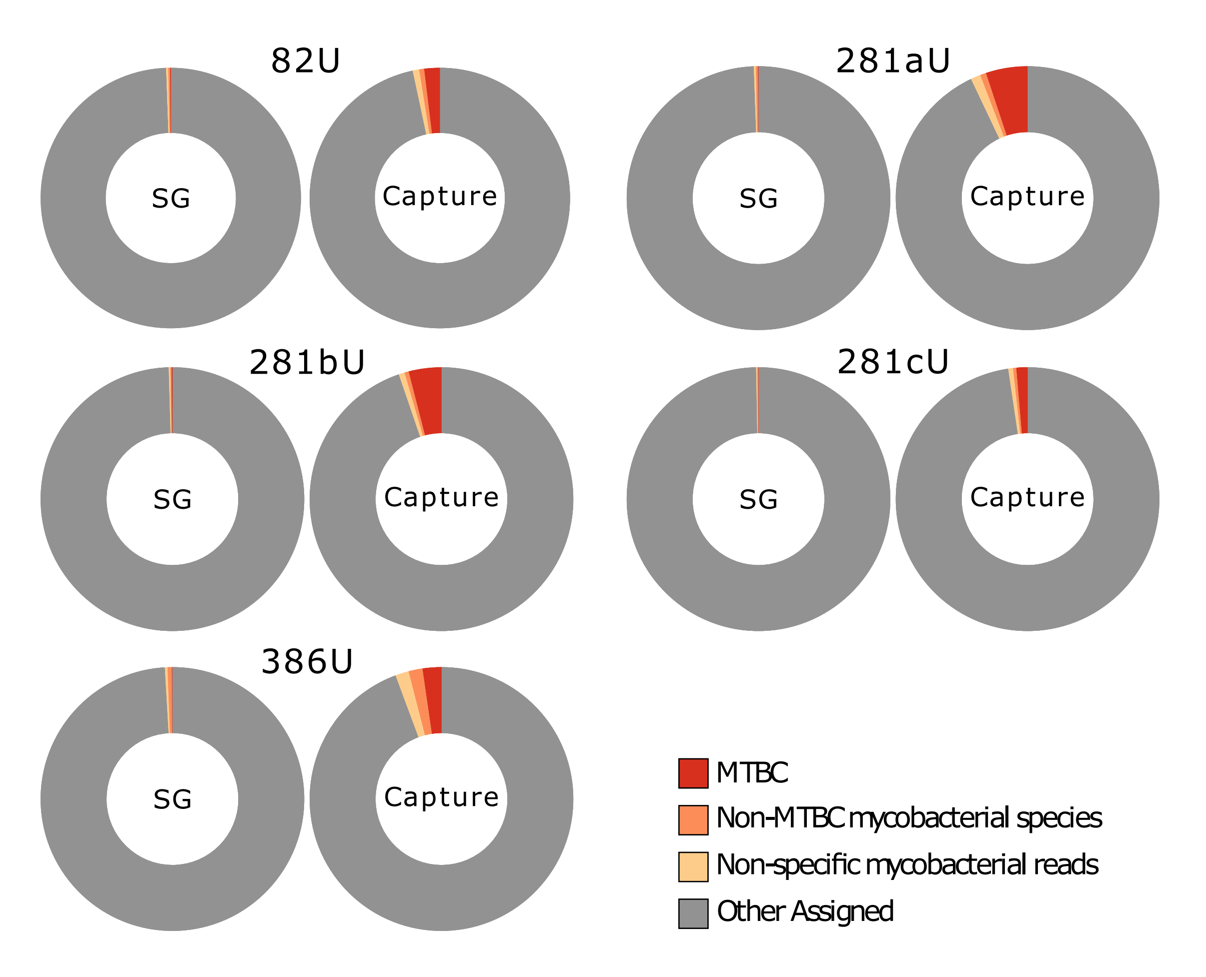

Supplementary Figure 1. MALT analysis of mycobacterial read proportions in sample libraries before and after capture. Shotgun and capture data for the UDG treated libraries, from which whole genomes were generated, were analyzed with MALT using the full NCBI Nucleotide (nt) database (7th Dec. 2016). Pie charts displaying proportions of mycobacterial reads assigned by MALT amongst samples positive for MTBC DNA before and after in-solution capture. The proportions shown are based on the reads that could be assigned by MALT to a taxon included in the database when using an 85% identity filter. SG= un-enriched shotgun data, Capture = data after whole-genome enrichment.

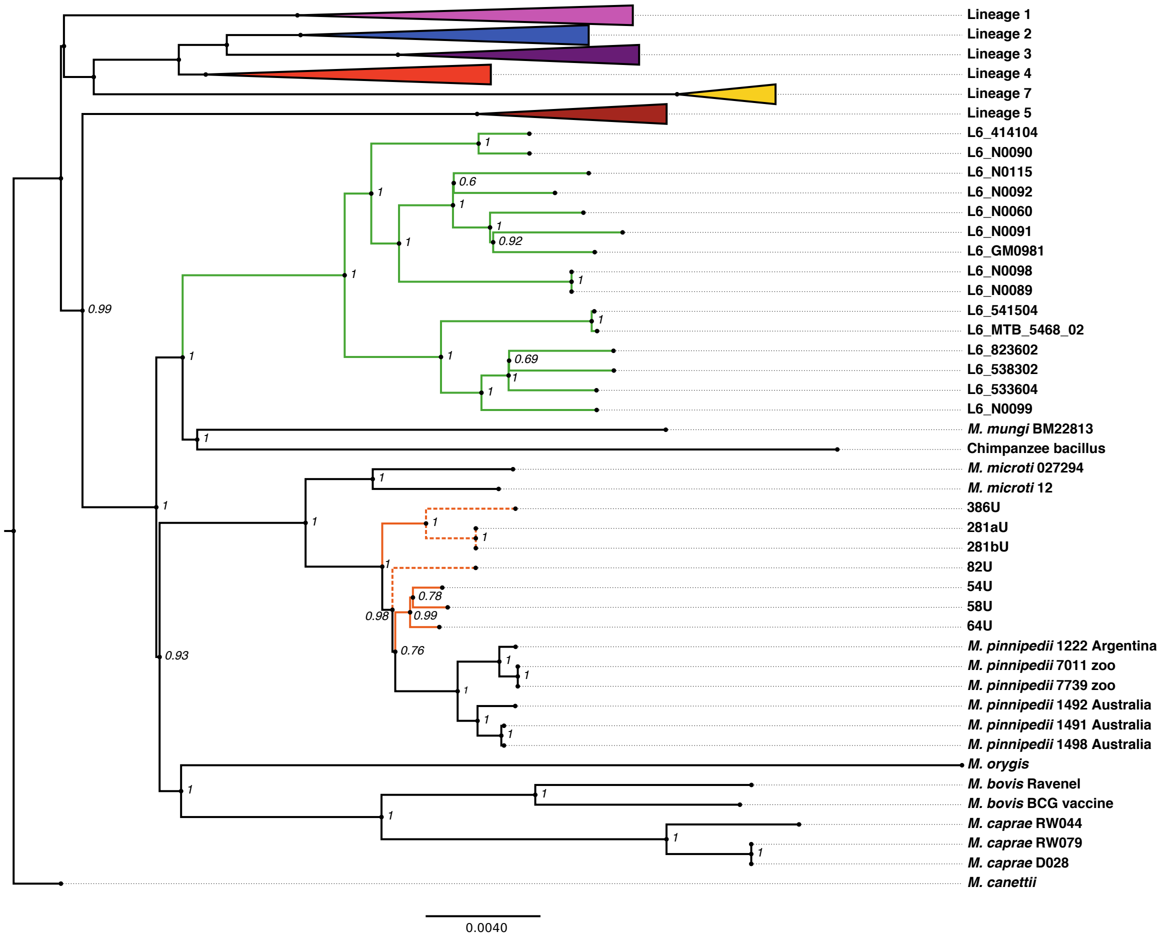

Supplementary Figure 2. Neighbour joining MTBC phylogeny with 281aU and 281bU separated. The phylogenetic tree was constructed using the full dataset of 267 genomes including the seven ancient genomes where the genomic data from 281aU and 281bU are separated. Ancient genomes are highlighted in orange. Human-adapted lineages 1-5 and 7 have been collapsed. The tree was constructed by excluding all missing and ambiguous data, using 1000 bootstrap replicates and is based on 10,765 positions out of a possible 44,304. Our three ancient genomes fall together with other ancient Peruvian genomes within the *M. pinnipedii* clade; due to their artificially longer branch lengths these branches are marked with a dashed line.

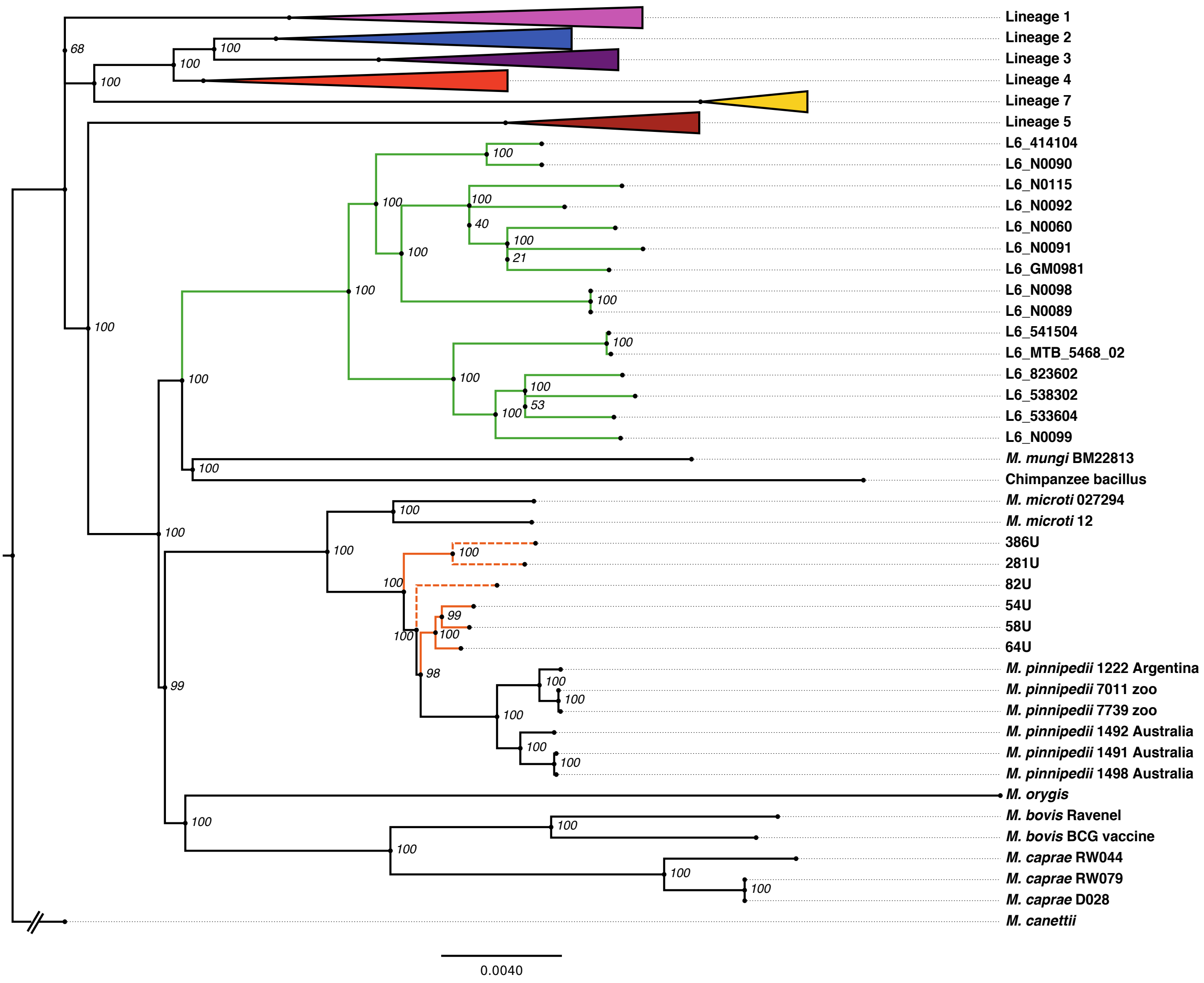

Supplementary Figure 3. Maximum Likelihood MTBC phylogeny. The phylogenetic tree was constructed using the full dataset of 266 genomes including the six ancient genomes, which are highlighted in orange. Human-adapted lineages 1-5 and 7 have been collapsed. The tree was constructed by excluding all missing and ambiguous data, using 1000 bootstrap replicates, and is based on 14,263 positions out of a possible 44,273. Our three ancient genomes fall together with other ancient Peruvian genomes within the *M. pinnipedii* clade; due to their artificially longer branch lengths these branches are marked with a dashed line.

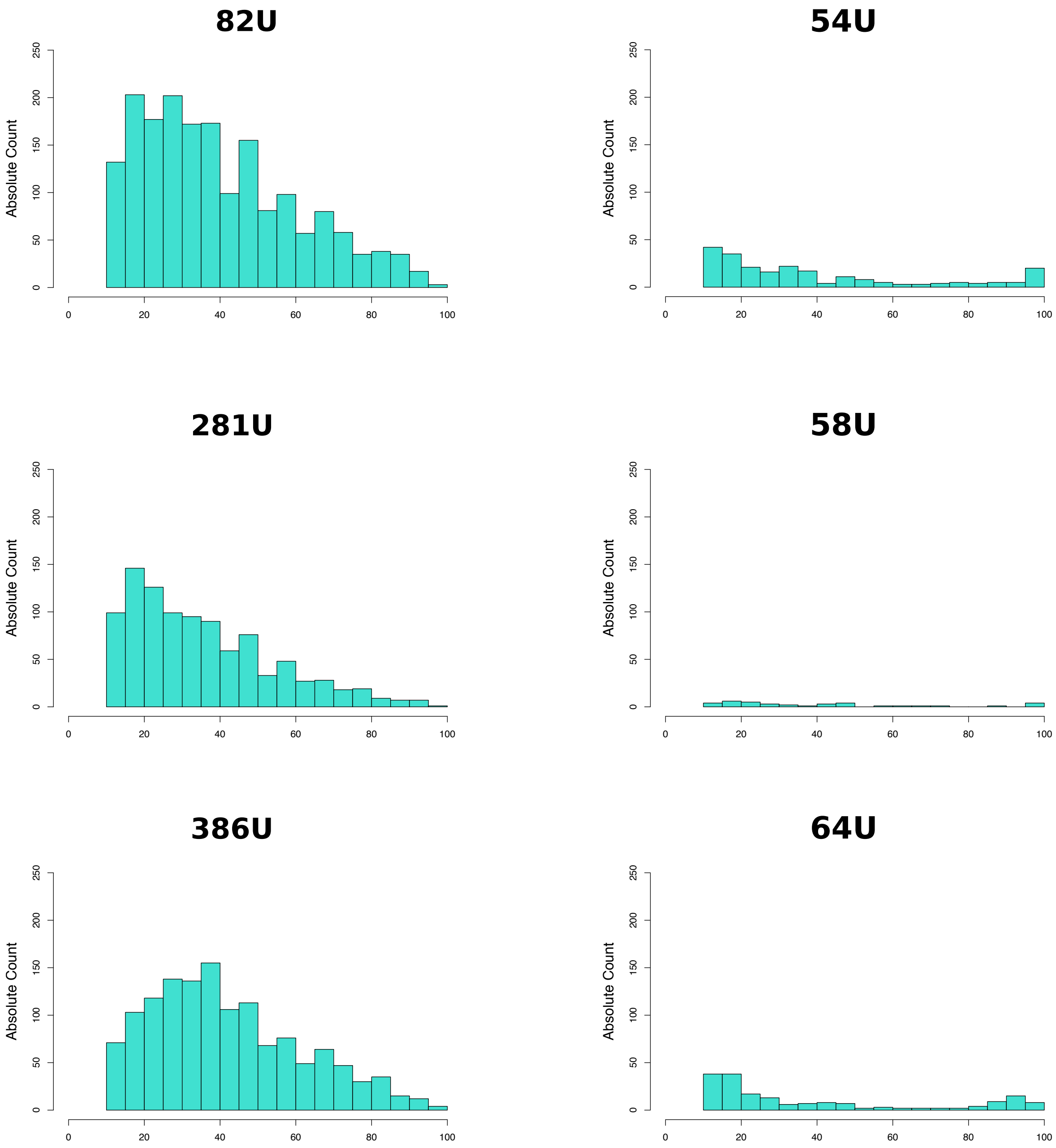

Supplementary Figure 4. **Histograms of SNP allele frequency distributions for ancient *M. pinnipedii* genomes**. The x-axis shows the SNP allele frequencies as a percentage. All variants where the SNP allele frequency is higher than 10% and lower than 100% are shown.

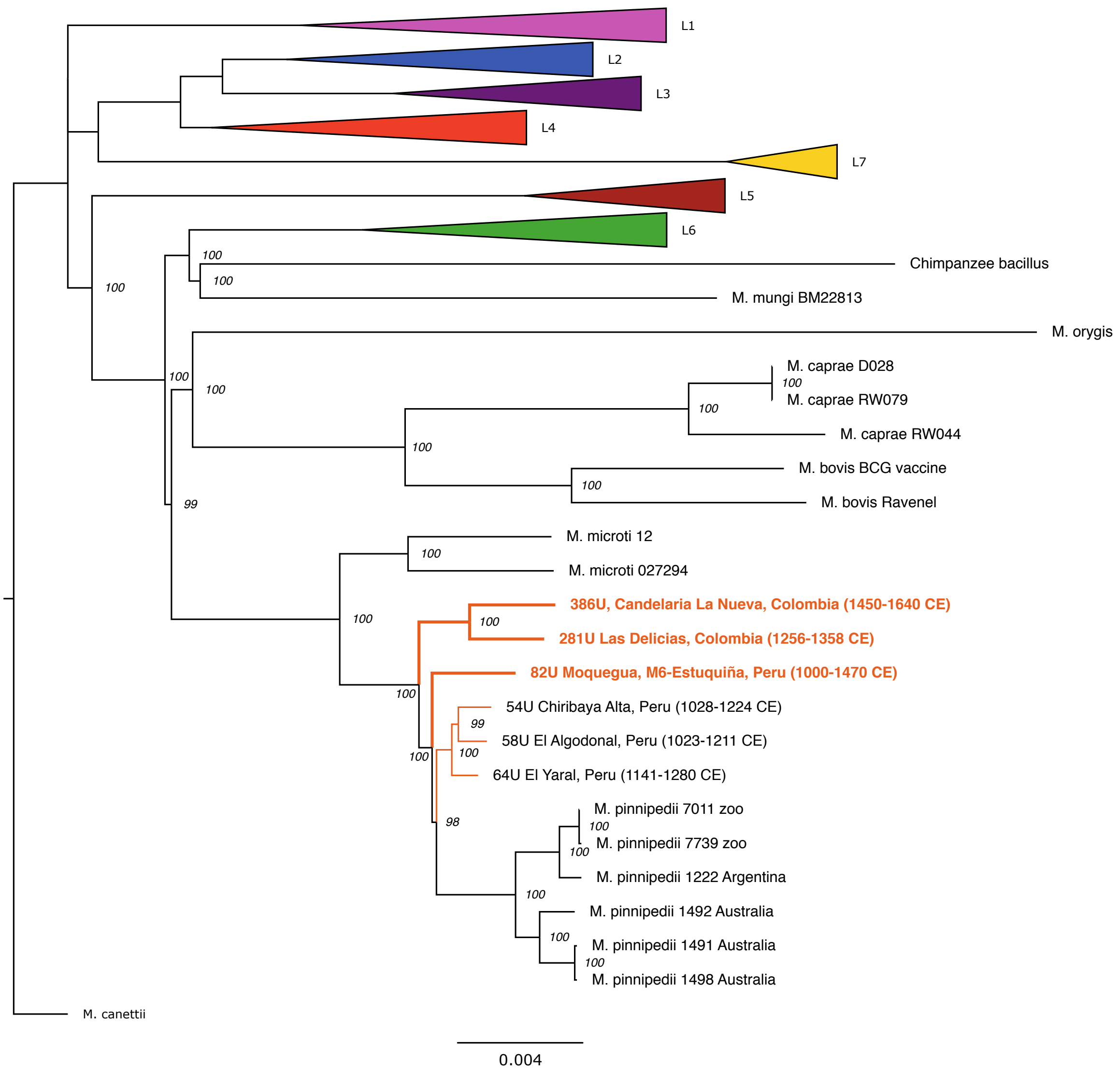

Supplementary Figure 5. Maximum Likelihood MTBC phylogeny after filtering of spurious reads from 82U, 281U and 386U. The phylogenetic tree was constructed using the full dataset of 266 genomes including the six ancient genomes, which are highlighted in orange. Human-adapted lineages 1-5 and 7 have been collapsed, in addition to human associated strains in lineage 6. The tree was constructed by excluding all missing and ambiguous data, using 1000 bootstrap replicates, and is based on 14,262 positions out of a possible 44,235.

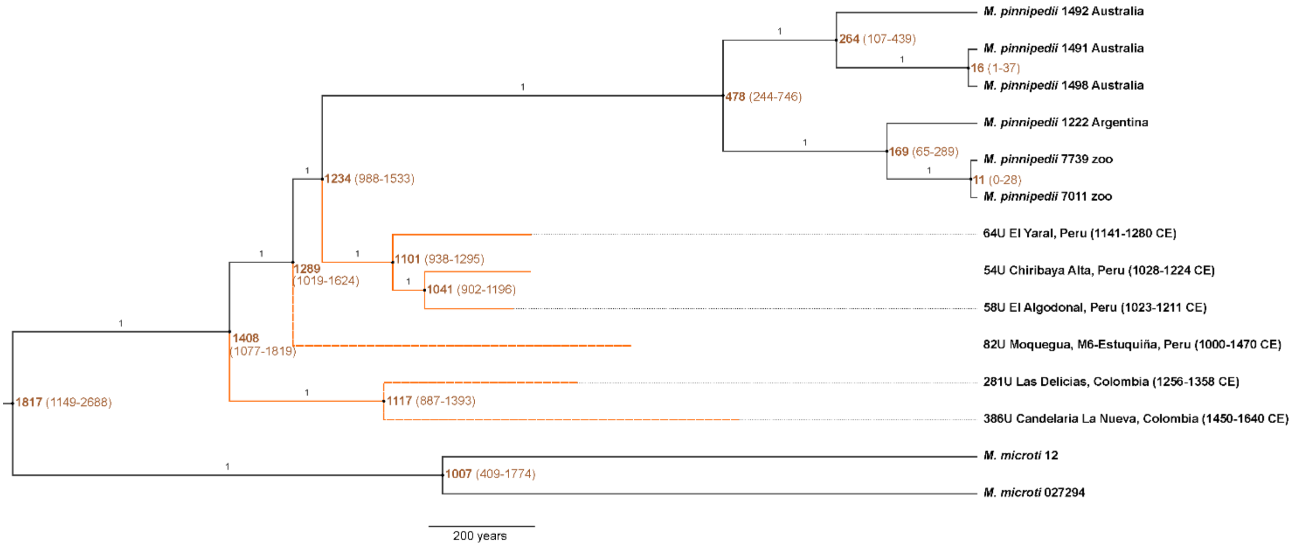

Supplementary Figure 6. Maximum clade credibility tree of *M. pinnipedii* and *M. microti* strains. Nodes are labelled with mean divergence time in years before present (YBP) with the 95% Highest Posterior Density (HPD) interval given in parentheses. Branches are labelled with their corresponding posterior probabilities. Branches leading to the ancient human *M. pinnipedii* genomes are marked in orange; dashed branches indicate the three genomes from this study (82U, 281U, and 386U).

Supplementary References

1 Rice, D. S., Conrad, G. W. & Buikstra, J. in *Trabajos Anqueologicos en Moquegua, Peru* Vol. 3 (eds Luis K. Watanabe, Michael E. Moseley, & Fernando Cabieses) Ch. 23, 39-93 (Programa Contisuyu del Museuo Peruana de Sciences de la Salud, Southern Peru Copper Corporation, 1990).

2 Clark, N. R. *The Estuquiña textile tradition: cultural patterning in late prehistoric fabrics, Moquegua, far southern Peru*, (1993).

3 Conrad, G. W. in *Domestic Architecture, Ethnicity, and Complementarity in the South-Central Andes* (ed Mark S. Aldenderfer) 55-65 (University of Iowa, 1993).

4 Stanish, C. & Rice, D. S. in *Ecology, Settlement and History in the Osmore Drainage, Peru* (eds Don S Rice, Charles Stanish, & Phillip R Scarr) (BAR International Series, 1989).

5 Sharratt, N. Steering Clear of the Dead: Avoiding Ancestors in the Moquegua Valley, Peru. *American Anthropologist* **119**, 645-661, doi:10.1111/aman.12944 (2017).

6 Williams, S. A. *The Skeletal Biology of Estuquiña: A Late Intermediate Period Site in Southern Peru*, Northwestern University, (1990).

7 Buikstra, J. E. & Williams, S. R. in *Human palaeopathology: Current syntheses and future options* (eds Donald Ortner & A. C. Aufderheide) 161-172 (Smithsonian Institution Press, 1991).

8 Bürgi, P. T. *et al.* in *Ecology, Settlement and History in the Osmore Drainage, Peru* Vol. 545(ii) (eds Don S. Rice, Charles Stanish, & Phillip R. Scarr) Ch. 18, (BAR International Series, 1989).

9 deFrance, S. in *ICAZ, Fish Remains Working Group Meetings.* (Portland, OR, 2019).

10 Diez de San Miguel, G., Espinoza Soriano, W., GutiÈrrez Flores, P., Murra, J. V. & Jay, I. K. R. C. *Visita hecha a la Provincia de Chucuito por Garci Diez de San Miguel en el año 1567. Versión paleográfica de la Visita y una biografía del visitador por Waldemar Espinoza Soriano. Padrón de los mil indios ricos de la Provincia de Chucuito en el año 1574.* (Casa de la Cultura del Per˙, 1964).

11 Cifuentes Toro, J. A. Proyecto de rescate arqueológico de la avenida Villavicencio: barrio Candelaria la Nueva, Bogotá. (Bogotá : ICANH, 1987).

12 Kurella, D. in *Chiefdoms and Chieftaincy in the Americas* (ed Elsa M. Redmond) 189-216 (University Press of Florida, 1998).

13 Cárdenas Arroyo, F. Reconstrucción química de la paleodieta en restos arqueológicos humanos del territorio muisca. *Revista Eres. Serie de arqueología,* **5**, 71-82 (1994).

14 Garcia, J. L. *The Foods And Crops Of The Muisca: A Dietary Reconstruction Of The Intermediate Chiefdoms Of Bogota (bacata) And Tunja (hunza), Colombia* Master of Arts (M.A.) thesis, University of Central Florida, (2012).

15 Enciso Ramos, B. E. Arqueología de rescate, en el barrio las Delicias, Bogotá. *Revista Colombiana de Antropología* **28**, 155-160, doi:10.22380/2539472X.1933 (1991).

16 Cardenas Arroyo, F. Paleodieta y Paleodemografia en Poblaciones Arqueologicas Muiscas (Sitios Las Delicias y Candelaria). *Revista Colombiana de Antropologia* **XXX** (1993).

17 Instituto Colombiano de Antropologia e Historia (ICANH). *Consultar la colección ósea del ICANH (en el Museo Nacional de Colombia)*, <http://www.icanh.gov.co/servicios_ciudadano/tramites_servicios/servicios/laboratorio_arqueologia/4550&download=Y> (

18 Instituto Colombiano de Antropologia e Historia (ICANH). *Registro Materiales Arqueológicos*,

<http://www.icanh.gov.co/nuestra_entidad/grupos_investigacion/arqueologia/parques_asociados/5151> (
19 Delgado, M. Stable isotope evidence for dietary and cultural change over the Holocene at the Sabana de Bogotá region, Northern South America. *Archaeological and Anthropological Sciences* **10**, 817-832 (2016).
20 Zajec, D. N. Paleodemografía de la población Muisca del sitio Candelaria la Nueva: informe final de trabajo de campo. (Bogotá : Universidad de los Andes, 1989).
21 Therrien, M. & Enciso, B. Avances de investigación: una re-investigacion arqueológica en la sabana de Bogotá. *Boletín Museo del Oro* **31**, 130-131 (1992).
22 Jara, N. P. Aplication of authenticity criteria in mitochondrial studies on archaic bone remains from a prehispanic Muisca population. *Colombia Médica* **41** (2010).
