# Supplementary material for: Geographically dispersed zoonotic tuberculosis in pre-contact South American human populations
Source: Nat Commun. 2022 Mar 7;13:1195. doi: 10.1038/s41467-022-28562-8 (PMC8901693; doi:10.1038/s41467-022-28562-8)
Supplement: Supplementary file 3 — Description of Additional Supplementary Files [file 41467_2022_28562_MOESM3_ESM.pdf]

Description of Additional Supplementary Files

File Name: Supplementary Data 1
Description: Overview of samples and corresponding qPCR and gene capture screening results.

File Name: Supplementary Data 2
Description: Overview of mapping statistics for UDG treated shotgun and non-UDG and UDG treated capture data to the MTBC_anc reference.

File Name: Supplementary Data 3
Description: Taxon table generated in MEGAN6 based on the MALT Full Nucleotide database results from the UDG treated shotgun and capture data using an 85% identity filter and minimum support of 1.

File Name: Supplementary Data 4
Description: Values used to evaluate the level of MTBC reads in UDG-treated shotgun and capture data relative to non-target DNA.

File Name: Supplementary Data 5
Description: Taxon table generated in MEGAN6 based on the MALT Full Nucleotide database results from the non-UDG treated shotgun data using an 85% identity filter and minimum support of 1.

File Name: Supplementary Data 6
Description: Taxon table generated in MEGAN6 based on the MALT Full Nucleotide database results from the non-UDG treated shotgun data using an 95% identity filter and minimum support of 1.

File Name: Supplementary Data 7
Description: Comparative analysis of MALT results on non-UDG treated shotgun data for all six ancient samples.

File Name: Supplementary Data 8
Description: Taxon table generated in MEGAN6 based on the MALT Full Nucleotide database results for the captured extraction and library negative controls using 95% identity filter and minimum support of 1.

File Name: Supplementary Data 9
Description: Damage pattern mapping-test. Mapping statistics for filtered non-UDG reads mapped to the MTBC_anc.

File Name: Supplementary Data 10
Description: Mapping statistics of non-UDG treated capture data to the human genome (hg19).

File Name: Supplementary Data 11
Description: Radiocarbon dates and stable isotope data generated as part of this study (Beta Analytic Inc.).

File Name: Supplementary Data 12
Description: Information about strains used in phylogenetic and SNP analyses.

File Name: Supplementary Data 13
Description: Overview of SNPs that occur in at least one of the ancient or modern *M. pinnipedii* genomes.

File Name: Supplementary Data 14
Description: Coordinates and number of reads supporting the deletion breakpoints for regions of difference.

File Name: Supplementary Data 15
Description: Sequences and optimized final concentrations of qPCR primers and probes used for MTBC screening assays.
