# Supplementary material for: Geographically dispersed zoonotic tuberculosis in pre-contact South American human populations
Source: Nat Commun. 2022 Mar 7;13:1195. doi: 10.1038/s41467-022-28562-8 (PMC8901693; doi:10.1038/s41467-022-28562-8)
Supplement: Supplementary file 5 — Reporting Summary [file 41467_2022_28562_MOESM5_ESM.pdf]

nature research

Corresponding author(s): Johannes Krause; Anne C. Stone; Kirsten I. Bos

Last updated by author(s): Dec 12, 2021

Reporting Summary

Nature Research wishes to improve the reproducibility of the work that we publish. This form provides structure for consistency and transparency in reporting. For further information on Nature Research policies, see Authors & Referees and the Editorial Policy Checklist .

Statistics

For all statistical analyses, confirm that the following items are present in the figure legend, table legend, main text, or Methods section.

n/a	Confirmed	
☒	☐	The exact sample size (*n*) for each experimental group/condition, given as a discrete number and unit of measurement
☒	☐	A statement on whether measurements were taken from distinct samples or whether the same sample was measured repeatedly
☒	☐	The statistical test(s) used AND whether they are one- or two-sided<br>*Only common tests should be described solely by name; describe more complex techniques in the Methods section.*
☒	☐	A description of all covariates tested
☒	☐	A description of any assumptions or corrections, such as tests of normality and adjustment for multiple comparisons
☒	☐	A full description of the statistical parameters including central tendency (e.g. means) or other basic estimates (e.g. regression coefficient) AND variation (e.g. standard deviation) or associated estimates of uncertainty (e.g. confidence intervals)
☒	☐	For null hypothesis testing, the test statistic (e.g. *F*, *t*, *r*) with confidence intervals, effect sizes, degrees of freedom and *P* value noted<br>*Give P values as exact values whenever suitable.*
☐	☒	For Bayesian analysis, information on the choice of priors and Markov chain Monte Carlo settings
☒	☐	For hierarchical and complex designs, identification of the appropriate level for tests and full reporting of outcomes
☒	☐	Estimates of effect sizes (e.g. Cohen's *d*, Pearson's *r*), indicating how they were calculated

Our web collection on statistics for biologists contains articles on many of the points above.

Software and code

Policy information about availability of computer code

Data collection	No software was used
Data analysis	All software used in this paper is open source and publicly available. Below is a list of computational tools used to analyze the genetic data presented in this paper.<br>SeqPrep v1.2 (https://github.com/jstjohn/SeqPrep)<br>Burrows-Wheeler Aligner (BWA) (v0.7.5 and v0.7.12)<br>SAMtools (v0.1.19)<br>Geneious R7<br>bcl2fastq (v.2.17)<br>EAGER pipeline (v.1.92.55)<br>AdapterRemoval (v. 2.2.0)<br>Dedup (v.0.12.2)<br>Megan ALignment Tool (MALT) (v. 0.3.8)<br>MEGAN6 community edition (v.6.12.3)<br>Picard tool (v. 1-140)<br>GATK (v. 3.5)<br>Integrated Genomics Viewer (IGV) v.2.8.0<br>MultiVCFanalyzer (v. 0.87) (https://github.com/alexherbig/MultiVCFAnalyzer)<br>MEGA 6<br>RAxML (v.8)<br>mapDamage 2.0<br>SnpEff (v. 3.1)<br>seqkit (v.0.11.0)

seqtk (v. 1.3) (https://github.com/lh3/seqtk)
BLASTN (https://blast.ncbi.nlm.nih.gov/Blast.cgi)
BEAST (v1.8.4)
TempEst (part of BEAST software package)
LogCombiner (part of BEAST software package)
TreeAnnotator (part of BEAST software package)
Tracer (v.1.7.1)
Figtree (http://tree.bio.ed.ac.uk/software/figtree/)

For manuscripts utilizing custom algorithms or software that are central to the research but not yet described in published literature, software must be made available to editors/reviewers. We strongly encourage code deposition in a community repository (e.g. GitHub). See the Nature Research guidelines for submitting code & software for further information.

Data

Policy information about availability of data

All manuscripts must include a data availability statement. This statement should provide the following information, where applicable:

- Accession codes, unique identifiers, or web links for publicly available datasets
- A list of figures that have associated raw data
- A description of any restrictions on data availability

All raw sequencing data for the MTBC-genome captured libraries which yielded the three M. pinnipedii genomes analyzed in this study (samples 82, 281, and 386) and captured negative control libraries have been deposited in the NCBI Sequence Read Archive under BioProject accession number PRJNA779792 (https://www.ncbi.nlm.nih.gov/sra/PRJNA779792). The final contaminant filtered BAM files used for variant calling are available on GitHub (https://github.com/ashildv/South_American_ancient_M.pinnipedii). Public datasets used in this manuscript include previously published MTBC genomes for which all accession numbers are provided in Supplementary Table 12, the human reference genome (hg19) available under NCBI GenBank accession code GCA_000001405.27 and the full NCBI Nucleotide collection (nt) database downloaded on 7th Dec. 2016 (https://ftp-trace.ncbi.nih.gov/blast/db/FASTA/).

Field-specific reporting

Please select the one below that is the best fit for your research. If you are not sure, read the appropriate sections before making your selection.

☒ Life sciences ☐ Behavioural & social sciences ☐ Ecological, evolutionary & environmental sciences

For a reference copy of the document with all sections, see nature.com/documents/nr-reporting-summary-flat.pdf

Life sciences study design

All studies must disclose on these points even when the disclosure is negative.

Sample size	In our study we have a sample size of 10 archaeological individuals/skeletal human remains that showed skeletal lesions consistent with prolonged tuberculosis infection. Bone samples were taken and screened for the presence of ancient tuberculosis DNA. Three of the ten individuals/samples were sufficiently positive for ancient tuberculosis DNA. Our initial sample size is 10, because we were given permission to take samples from the human remains of 9 individuals from different sites across Colombia by the Colombian Institute of Anthropology and History (ICANH). Additionally, we have one sample (82) from Peru that was screened as part of our previous study (Bos et al. 2014, Nature), which was positive for ancient tuberculosis DNA, but it was not pursued for whole genome capture for tuberculosis at that time. This sample was therefore included in this study for whole genome capture, thus increasing the sample size to 10.
Data exclusions	Data generated from our ancient samples that were not positive for tuberculosis DNA via qPCR and/or gene capture screening approaches were excluded from further analyses. After whole genome capture one of three captured samples for individual 281 (samples 281cU) was excluded from further analysis due to the low amount of on-target tuberculosis DNA and high environmental mycobacterial background. The two other captured samples for individual 281 (281aU and 281bU) yielded higher amounts of on-target DNA and were sufficient for whole-genome re-construction.
Replication	qPCR experiments were replicated in triplicate. Experiment replication was not relevant to any other part of our study. Our 3 ancient tuberculosis genomes were verified through our recovery of genome-wide data at a minimum depth of 10-fold average coverage in all cases, these genomes were further verified through phylogenetic placement.
Randomization	Randomization was not relevant because our study does not make use of experimental groups.
Blinding	Blinding was not relevant because our study does not make use of experimental groups.

Reporting for specific materials, systems and methods

We require information from authors about some types of materials, experimental systems and methods used in many studies. Here, indicate whether each material, system or method listed is relevant to your study. If you are not sure if a list item applies to your research, read the appropriate section before selecting a response.

Materials & experimental systems

n/a	Involved in the study
☒	☐ Antibodies
☒	☐ Eukaryotic cell lines
☒	☐ Palaeontology
☒	☐ Animals and other organisms
☒	☐ Human research participants
☒	☐ Clinical data

Methods

n/a	Involved in the study
☒	☐ ChIP-seq
☒	☐ Flow cytometry
☒	☐ MRI-based neuroimaging
